# 860. Impact of Neurologic Complications on Burn Patient Outcomes

**DOI:** 10.1093/jbcr/irag033.330

**Published:** 2026-04-06

**Authors:** Sonya Baidar, Kathleen S Romanowski, Jason Heard, Soman Sen, Tina L Palmieri

**Affiliations:** Shriners Children's Northern California, Elk Grove, CA; Shriners Children's - Northern California, Sacramento, CA; Shriners Children's - Northern California, Sacramento, CA; Shriners Children's - Northern California, Sacramento, CA; University of California Davis Health, Shriners Children’s - Northern California, Sacramento, CA

## Abstract

**Introduction:**

Although age, burn size, and inhalation injury are the primary drivers of outcomes after burn injury, other complications can also influence outcome. We hypothesized that patients with neurological complications would have worse outcomes in terms of survival, ventilator days, and length of stay (LOS).

**Methods:**

This single center retrospective analysis of the National Burn Repository between 2015-2022 compared outcomes for patients with and without neurologic complications admitted to our facility. We collected demographic data (age, sex, race/ethnicity, marital status), injury characteristics (total burn surface area, inhalation injury), and outcomes (LOS, ventilator days, ICU stay, and survival. We used chi-square (mortality, sex, inhalation injury), T-test (age, race), and Wilcox Rank-Sum tests (burn size, length of stay) with *p*<.05 considered significant.

**Results:**

A total of 710 patients were identified with 179 neurologic conditions (NC), and 2063 patients without neurologic conditions (control group-CG). CG patients were on average older than the NC group (47.6 ± 12 vs 45.0 ± 31.1 years old), had smaller burns (11.8 ± 4.9% vs. 12.4 ± 0.7%), and had a shorter ICU stay (10.4 ± 0.7 vs 11.5 ± 9.2 days, *p*<.05). There was no difference in mortality (5% in CG, 4.2% in NC), or hospital LOS (16.4 ± 3.5 days CG vs. 15.8 ± 24.7 days).

**Conclusions:**

Patients who develop neurologic complications have longer ICU stays and consume more resources. Early identification and treatment of neurologic complications may mitigate these effects.

**Applicability of Research to Practice:**

By better understanding the effects of neurologic complications, we can proactively intervene to identify and mitigate them.

**Funding for the study:**

N/A.

